# Synthetic oleanane triterpenoids suppress *MYB* oncogene activity and sensitize T-cell acute lymphoblastic leukemia cells to chemotherapy

**DOI:** 10.3389/fonc.2023.1126354

**Published:** 2023-04-03

**Authors:** Paloma Tejera Nevado, Tajana Tešan Tomić, Ali Atefyekta, André Fehr, Göran Stenman, Mattias K. Andersson

**Affiliations:** Sahlgrenska Center for Cancer Research, Department of Pathology, University of Gothenburg, Gothenburg, Sweden

**Keywords:** *MYB*, acute lymphoblastic leukemia, oleanane triterpenoid, bardoxolone methyl, omaveloxolone

## Abstract

T-cell acute lymphoblastic leukemia (T-ALL) is an aggressive hematologic malignancy with poor prognosis. The *MYB* oncogene encodes a master transcription factor that is activated in the majority of human T-ALLs. In the present study, we have performed a large-scale screening with small-molecule drugs to find clinically useful inhibitors of *MYB* gene expression in T-ALL. We identified several pharmacological agents that potentially could be used to treat MYB-driven malignancies. In particular, treatment with the synthetic oleanane triterpenoids (OTs) bardoxolone methyl and omaveloxolone decreased *MYB* gene activity and expression of MYB downstream target genes in T-ALL cells with constitutive *MYB* gene activation. Notably, treatment with bardoxolone methyl and omaveloxolone led to a dose-dependent reduction in cell viability and induction of apoptosis at low nanomolar concentrations. In contrast, normal bone marrow-derived cells were unaffected at these concentrations. Bardoxolone methyl and omaveloxolone treatment downregulated the expression of DNA repair genes and sensitized T-ALL cells to doxorubicin, a drug that is part of the standard therapy of T-ALL. OT treatment may thus potentiate DNA-damaging chemotherapy through attenuation of DNA repair. Taken together, our results indicate that synthetic OTs may be useful in the treatment of T-ALL and potentially also in other MYB-driven malignancies.

## 1 Introduction

T-cell acute lymphoblastic leukemia (T-ALL) is an aggressive hematologic malignancy characterized by abnormal expansion of transformed lymphoid progenitor cells in the bone marrow, blood, and extramedullary sites (1, 2). It accounts for approximately 25% of ALL cases in adults and 10-15% of cases in children. Even though risk-based stratification and dose-intensification therapy have led to marked improvements in survival of pediatric patients, the prognosis for adults remains poor, particularly for older patients (2). Less than half of adults diagnosed with T-ALL survive more than five years (3). Thus, development of improved therapies for this disease is needed.

The *MYB* oncogene is activated in the majority of T-ALLs (4, 5). It encodes a pioneer master transcription factor that binds to super-enhancers and regulates chromatin accessibility and genes involved in cell division and maturation (5, 6). In T-ALL, *MYB* gene activation leads to blocked differentiation and increased proliferation of neoplastic lymphoid progenitor cells, suggesting that the gene is a potential therapeutic target in this disease (4, 7). The *MYB* gene is activated by various molecular mechanisms in T-ALL. *MYB* is activated by the TAL1 complex that is overexpressed in up to 60% of T-ALL (8) and is a downstream target of MLL-fusions (9). In 10-15% of T-ALLs, *MYB* is activated by gene duplication or gene fusion (7, 10, 11). Furthermore, potentially activating hot spot mutations in the 5´-part of *MYB* were recently described in T-ALL (11). We and others have previously shown that the MYB protein and its downstream effectors can be successfully targeted in acute myeloid leukemia (AML) (12–24). However, very few MYB inhibitors with therapeutic effects in T-ALL have been identified (25). Given that MYB is an oncogenic driver in the majority of T-ALLs and primarily regulated at the transcriptional level (26), we decided to perform a large-scale drug screening with small-molecule agents to identify inhibitors of *MYB* gene expression. We hypothesized that suppression of MYB might improve the treatment of patients with this aggressive disease that are refractory to the conventional therapies currently used. We now show that synthetic oleanane triterpenoids both inhibit MYB expression and sensitize T-ALL cells to chemotherapy.

## 2 Materials and methods

### 2.1 Cell culture

The T-ALL cell lines (27–29) MOLT-4 (ATCC no. CRL-1582), CCRF-CEM (ATCC no. CCL-119), P12-ICHIKAWA (DSMZ no. ACC-34), and RPMI-8402 (DSMZ no. ACC-290) were obtained from ATCC (Manassas, VA, USA) and DSMZ (Braunschweig, Germany). The cell lines have *MYB*-activation by gene duplication (7, 10). Cell identity was confirmed by STR-analysis and all cell lines were shown to be mycoplasma free prior to experiments. T-ALL cells were maintained in RPMI-1640 medium with GlutaMAX, 10% or 20% FBS, and 1% penicillin-streptomycin (Thermo Fisher Scientific, Waltham, MA, USA). Human mononuclear cells (Merck, Darmstadt, Germany) isolated from a healthy donor (HMCs) were maintained in Mononuclear cell medium (Merck) according to the instructions of the supplier. All cells were kept in a humidified incubator at 37°C and 5% CO_2_.

### 2.2 Chemical library, drug screening, and validation experiments

The L1100 Inhibitor Library, bardoxolone methyl (CDDO-Me, RTA 402), omaveloxolone (RTA 408), and doxorubicin were purchased from Selleck Chemicals (Houston, TX, USA). The IKK inhibitor VII and IKK-2 inhibitor IV were obtained from Merck. The KI696 peptide (30) was a kind gift from Dr. Volkan Sayin. Drugs were kept at -80°C or -20°C in stock solutions of 10 µM in DMSO and diluted to working concentrations in growth medium prior to experiments. For drug screening, 4,000 MOLT-4 or CCRF-CEM cells were seeded in V-bottom 96-well plates (Greiner Bio-One, Kremsmünster, Austria) and after 24 h treated with 1 µM of 768 small-molecule inhibitors from the Inhibitor Library (**Supplementary Table S1**). Cells were incubated with drugs, or DMSO as control, for 48 h whereafter they were washed with PBS and harvested using direct lysis (31) in 100 µl of 1 mg/ml BSA (Thermo Fisher Scientific). The screening was performed three times and cell plates were stored at -80°C until further analysis. For validation experiments, 2-2.5x10^5^ T-ALL cells were seeded in 24-well plates (RNA analysis) and 4x10^5^ cells were seeded in 6-well plates (protein analysis) and on the next day treated for 24 h with drugs or equal volumes of DMSO.

### 2.3 RNA isolation and cDNA synthesis

Total RNA was isolated using the RNeasy Micro-kit (Qiagen, Hilden, Germany) following the protocol of the manufacturer. RNA purity and concentration was measured with the NanoDrop ND-1000 spectrophotometer (Thermo Fisher Scientific). Reverse transcription was performed with the iScript cDNA synthesis kit (Bio-Rad, Hercules, CA, USA) according to instructions of the manufacturer. For drug screening, 2 µl of cell lysate were used in 10 µl cDNA reactions.

### 2.4 Western blot

Total protein was isolated using RIPA buffer supplemented with the Halt protease and phosphatase inhibitor (Thermo Fisher Scientific). Protein concentrations were measured with the DC protein assay (Bio-Rad). Protein expression was analyzed by Western blotting using the NuPAGE system (Thermo Fisher Scientific) with monoclonal antibodies to MYB (clone 1-1; Merck Millipore) and beta-actin (ab8226, Abcam, Cambridge, UK). Bands were visualized with horseradish peroxidase-conjugated secondary antibodies and chemiluminescent detection with the Supersignal West Femto Max Sensitivity Substrate (Thermo Fisher Scientific). Blots were scanned with an Amersham ImageQuant 800 imaging system (Cytiva, Marlborough, MA, USA). MYB protein expression was quantified using ImageJ v2.0.0-rc-43 and normalized with beta-actin expression.

### 2.5 Quantitative real-time PCR (qPCR)

qPCR was done with the AB 7500 Fast Real-Time PCR system using TaqMan gene expression assays (Thermo Fisher Scientific) for *MYB* (Hs00920556_m1), *CD34* (Hs00156373_m1), *CCNB1* (Hs00259126_m1), *MCM4* (Hs00381533_m1), *CDK1* (Hs00364293_m1), and the reference genes *UBC* (Hs01871556_s1) and *18S* (Hs99999901_s1). Relative gene expression was calculated by the ddCT method (32).

### 2.6 RNA-sequencing (RNA-seq)

RNA-seq was performed at Eurofins Genomics (Luxembourg City, Luxembourg) using the INVIEW Transcriptome Discover service. Library preparation was done with poly A enrichment, random primed strand-specific cDNA synthesis, adapter ligation, and adapter-specific PCR amplification. Libraries were subjected to Illumina paired-end sequencing (2x150 bps) with at least 30 million read pairs generated. Gene counts were extracted from FastQ files on the Uppsala Multidisciplinary Center for Advanced Computational Science (UPPMAX) cluster with FastQC/0.11.5, STAR v2.5.3a, Samtools v1.5, and FeatureCounts (Subread v1.5.2). Samples were analyzed with unsupervised hierarchical clustering using the *hclust* function in R and heatmaps were generated using R or Morpheus (Broad Institute). Differential gene expression was analyzed with DESeq2 v1.22.2. Gene ontology analysis was performed with ToppGene (33) and Gene set enrichment analysis (GSEA) (34) was done with GSEA v4.2.2 (Broad Institute).

### 2.7 Cell viability, cell cycle and apoptosis assays

For cell viability assays, 2,000 T-ALL cells were seeded in black 96-well plates (BD, Franklin Lakes, NJ, USA) and after 24 h treated with single or combinations of drugs. The AlamarBlue reagent (Thermo Fisher Scientific) was added to wells after 72 h and plates were analyzed with the VICTOR3 multilabel plate reader (PerkinElmer, Waltham, MA, USA). For apoptosis assays, 2,000 cells were seeded in Corning white 96-well plates (Corning, New York, USA) and treated with drugs after 24 h. The Caspase-Glo 3/7 Assay (Promega, Madison, WI, USA) was used to estimate apoptosis and luminescent signals were quantified on the VICTOR3. For cell cycle analysis, 8x10^5^ MOLT-4 cells were seeded in 6-well plates and on the next day treated for 24 h with drugs. Cells were supplemented with 10 μM BrdU during the last 4 h and then fixed and stained using the FITC BrdU Flow kit (BD). Stained cells were analyzed with an Accuri C6 flow cytometer (BD). All experiments were performed three times.

### 2.8 siRNA experiments

*MYB* siRNA-mediated knockdown was done with the Cell Line Nucleofector Kit L on the Amaxa Nucleofector II device (Cologne, Germany) using 2x10^6^ MOLT-4 cells according to the instructions provided by the manufacturer. Cells were electroporated with 1 µM Silencer Select *MYB* (s9109, s9110) or negative control siRNAs (Thermo Fisher Scientific). Total RNA and protein were isolated from transfected cells after 48 h. All experiments were done three times.

### 2.9 Statistical analysis

Unsupervised hierarchical clustering was done with R and overlap of differentially expressed genes was visualized with BioVenn (https://www.biovenn.nl) and analyzed with chi-square tests. Drug kernel density estimation plots were generated with the *density* function of the *stats* package of R (https://www.r-project.org). Correlation of treatment effects between T-ALL cell lines was estimated by Pearson correlation. Dose-response curves were generated and analyzed by nonlinear regression in Prism 9 (GraphPad Software, San Diego, CA, USA); IC_25_ and IC_50_ were defined as drug concentrations that reduced cell viability by 25% and 50%, respectively. For combination treatments, the definition of drug additivity and synergy was according to Bliss (35). Differences between experimentally measured effects of drug combinations and the expected effects calculated from single drug treatments were analyzed using independent samples t-test. Bar graph values are presented as mean ± SEM. Differences between groups in qPCR, cell cycle, and apoptosis analyses were evaluated with one-way ANOVA or independent samples t-test. All statistical tests were two-sided. A *P*-value of less than 0.05 was considered statistically significant.

## 3 Results

### 3.1 Large-scale drug screening identifies synthetic oleanane triterpenoids as inhibitors of *MYB* gene expression in T-ALL cells

**Figure 1:**
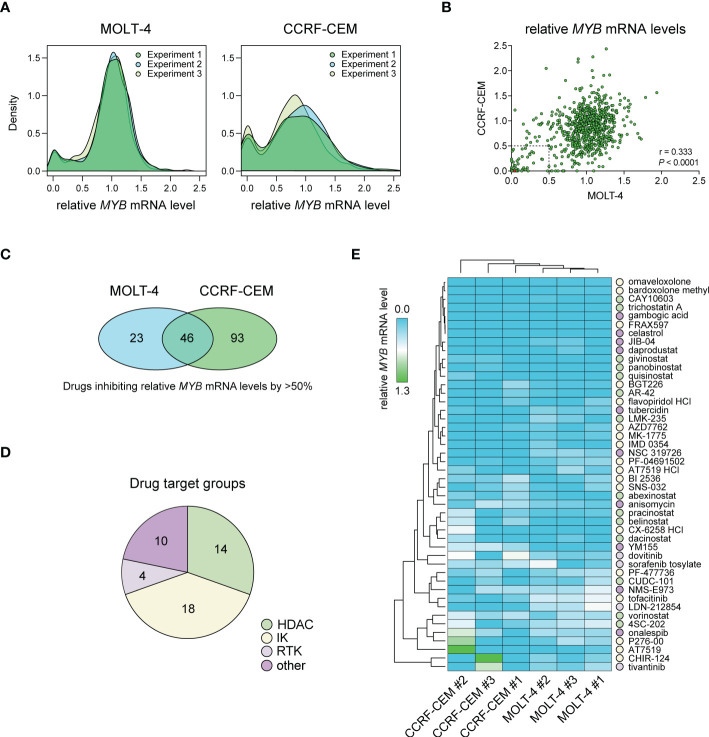
Drug screening for inhibitors of *MYB* gene expression in T-ALL cells with *MYB* gene activation. **(A)** Density plots showing treatment effects of 768 small-molecule inhibitors on *MYB* gene expression in two T-ALL cell lines. Data is derived from three independent experiments. **(B)** Pearson correlation analysis of treatment response for 768 inhibitors in two T-ALL cell lines. Dashed lines indicate thresholds for 50% *MYB* mRNA expression compared with DMSO treated control cells. The red dot shows bardoxolone methyl and omaveloxolone that had near identical effects on *MYB* expression in the screen. **(C)** Venn diagram showing the number of drugs reducing *MYB* gene expression with more than 50% in T-ALL cells. **(D)** Drug target groups of 46 inhibitors reducing *MYB* mRNA levels with more than 50% in two T-ALL cell lines. **(E)** Heat map and hierarchical clustering analyses showing the effects of 46 inhibitors on *MYB* gene expression. Drug targets with grouping as in D are indicated. IK, intracellular kinase; RTK, receptor tyrosine kinase; HDAC, histone deacetylase.

We performed a large-scale *in vitro* screening of two T-ALL cell lines, CCRF-CEM and MOLT-4, with *MYB*-activation using 768 small-molecule drugs from the L1100 Inhibitor Library (**Figure 1**, **Supplementary Table S1**). Cells were treated with 1 μM of inhibitors for 48 h after which relative *MYB* mRNA levels were analyzed by qPCR. Density plots of *MYB* gene expression following drug treatments showed similar distributions in three independent experiments for each cell line (**Figure 1A**). There was a significant (*P* < 0.0001) correlation between drug effects in the two cell lines (**Figure 1B**). A larger number of drugs reduced *MYB* mRNA levels in CCRF-CEM cells compared with MOLT-4 cells (**Figure 1A**). Further analysis showed that 46 drugs caused a greater than 50% decrease in *MYB* gene expression in both cases (**Figure 1C**). These agents included histone-deacetylase inhibitors (HDACi), intracellular kinase (IK) inhibitors, receptor tyrosine kinase (RTK) inhibitors, and other drugs with diverse targets (**Figure 1D**, **Supplementary Table S2**). Among these were drugs that were previously identified as inhibitors of *MYB* gene expression (AT7519, flavopiridol, givinostat, and vorinostat) (36–39) as well as an inhibitor of MYB protein activity (celastrol) (12). To validate our drug screen, we treated MOLT-4 cells with two of the identified HDACi (givinostat and panobinostat) and two of the cyclin-dependent kinase inhibitors (CDKi) (AT7519 and SNS-032) from the screen. All tested drugs significantly decreased *MYB* mRNA levels at nanomolar concentrations (**Supplementary Figure S1**). We next used hierarchical clustering and heat map analysis of *MYB* mRNA levels to investigate the potency of the inhibitors of *MYB* gene expression identified in our screen (**Figure 1E**). These analyses identified bardoxolone methyl and omaveloxolone, two related oleanane triterpenoids (OTs), as top hits in the screen.

### 3.2 Treatment of T-ALL cells with OTs leads to downregulation of MYB expression and downstream target genes

**Figure 2:**
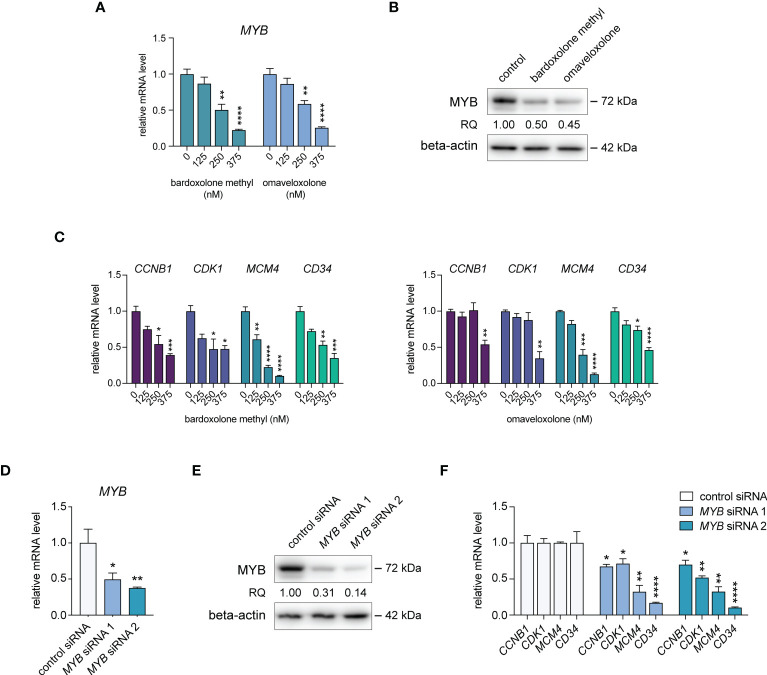
Treatment with oleanane triterpenoids (OTs) leads to decreased expression of MYB and its downstream target genes. **(A)** *MYB* mRNA expression in MOLT-4 cells treated with bardoxolone methyl and omaveloxolone for 24 h. **(B)** MYB protein expression in MOLT-4 cells treated for 24 h with 250 nM OTs. **(C)** Expression of MYB downstream target genes in MOLT-4 cells treated as in **(A, D)** Knockdown of *MYB* mRNA in MOLT-4 cells after treatment with 1 µM of *MYB* siRNAs for 48 h. **(E)** MYB protein expression in MOLT-4 cells treated with *MYB* siRNAs as in **(D, F)** Expression of MYB downstream target genes after *MYB* knockdown as in D and **(E)** Error bars indicate standard error of the mean. One-way ANOVA test (* *P* < 0.05; ** *P* < 0.01; *** *P* < 0.001; **** *P* < 0.0001). Data shown represents one of three independent experiments. RQ, relative quantification of MYB protein levels normalized with beta-actin expression.

To confirm the effects of OT treatment on *MYB* gene expression in T-ALL cells, we treated MOLT-4, CCRF-CEM, P12-ICHIKAWA, and RPMI-8402 cells with nanomolar concentrations of bardoxolone methyl or omaveloxolone for 24 h. This treatment led to a dose-dependent decrease in *MYB* mRNA levels in all T-ALL cell lines tested (**Figure 2A**, **Supplementary Figure S2**). A corresponding reduction in MYB protein levels after OT treatment was confirmed by immunoblotting (**Figure 2B**, **Supplementary Figure S3A**). To investigate the biological consequences of MYB suppression in OT treated cells, we analyzed the expression of known MYB target genes involved in cell cycle regulation and leukemic stem cell function. The expression of *CCNB1*, *CDK1*, *MCM4*, and *CD34* were significantly downregulated in MOLT-4 cells with OT treatment (**Figure 2C**). To verify that these genes are regulated by MYB in T-ALL cells, we knocked down *MYB* mRNA and protein levels in MOLT-4 cells using siRNAs (**Figures 2D, E**, **Supplementary Figure S3B**). Analogous to treatment with OTs, transfection with *MYB* siRNAs resulted in downregulation of *CCNB1*, *CDK1*, *MCM4*, and *CD34* (**Figure 2F**). Our results indicate that these genes are MYB targets in T-ALL that are downregulated following OT treatment.

Both bardoxolone methyl and omaveloxolone inhibit NF-κB (40, 41), a signaling pathway that has been shown to positively regulate *MYB* transcription in murine erythroleukemia cells (42, 43). To test whether NF-κB signaling is involved in *MYB* gene regulation also in human T-ALL cells, we treated MOLT-4 cells with two IKK-1 and IKK-2 inhibitors and measured *MYB* mRNA levels by qPCR (**Supplementary Figure S4A**). Neither of these inhibitors decreased *MYB* gene expression, which may indicate that OTs downregulate *MYB* gene activity independently of NF-κB signaling in T-ALL cells. Similarly, treatment with the cell-penetrating NRF2-activating peptide KI696 (30, 44) did not affect *MYB* gene expression in T-ALL cells (**Supplementary Figure S4B**), suggesting that OTs target other pathways than NF-κB and NRF2 to regulate the *MYB* gene in T-ALL cells.

### 3.3 RNA-seq reveals molecular signatures associated with a reversed MYB-driven transcriptional program and induction of apoptosis in OT treated T-ALL cells

**Figure 3:**
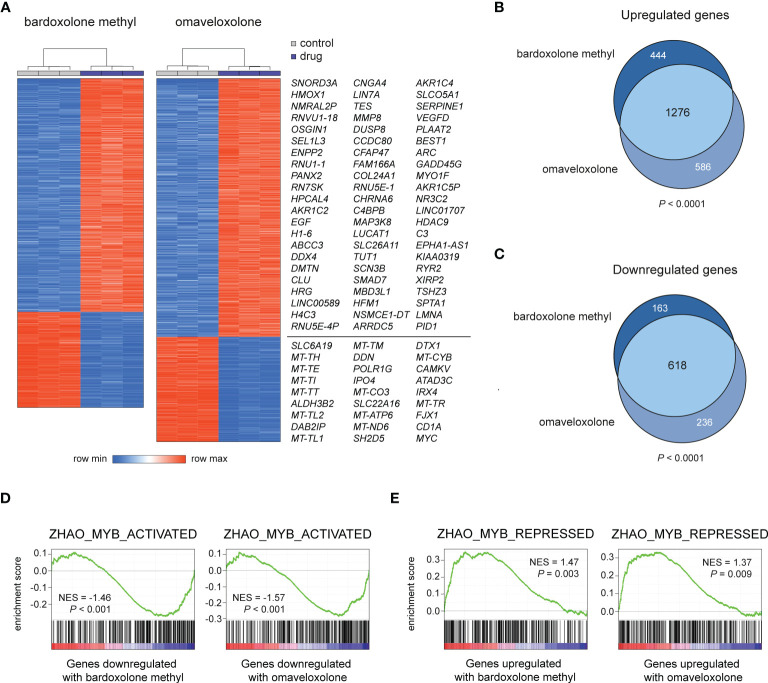
RNA-seq analysis of OT treated T-ALL cells. **(A)** Hierarchical clustering and heatmap analysis of global gene expression data from MOLT-4 cells treated for 24 h with 375 nM OTs. The most up- and downregulated genes by both drugs are shown to the right. **(B, C)** Venn diagrams of up- and downregulated genes in OT treated T-ALL cells. The significance of overlap was estimated by chi-square tests. **(D, E)** Gene set enrichment analysis of MYB-activated and repressed genes in OT treated T-ALL cells. NES, normalized enrichment score.

**Figure 4:**
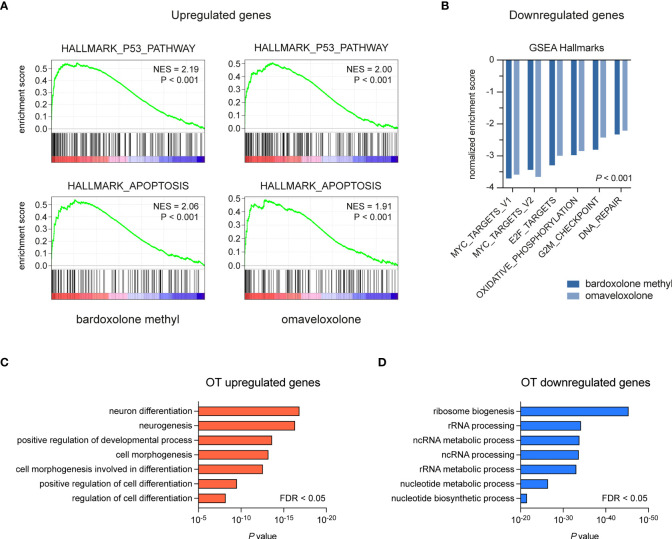
Gene set enrichment and gene ontology analysis of OT treated T-ALL cells. **(A, B)** Gene set enrichment analysis of pathways and gene sets affected by OT treatment in MOLT-4 cells. **(C, D)** Gene ontology analysis of up- and downregulated genes following OT treatment. NES, normalized enrichment score. FDR, false-discovery rate.

To study the effects of OT treatment on critical cellular processes in T-ALL cells, we performed RNA-seq analysis of MOLT-4 cells treated with 375 nM bardoxolone methyl or omaveloxolone for 24 h. Global gene expression analysis showed that OT treated cells clustered separately from control cells (**Figure 3A**). There was a significant overlap of differentially regulated genes between the two drugs (**Figures 3A–C**). To investigate if OT treatment affects the MYB-driven transcriptional program in T-ALL cells, we used GSEA to compare our RNA-seq data with a previously published dataset (45) of direct MYB target genes in hematopoietic progenitor cells (**Figures 3D, E**). OT treatment downregulated genes that are normally activated by MYB (e.g. *MYC*, *TCF7*, *MAT2A*) and upregulated known MYB-repressed genes (e.g. *CEBPB*, *IL17RA*, *BCL6*). Thus, our findings indicate that treatment with bardoxolone methyl and omaveloxolone can reverse the MYB-driven transcriptional program in T-ALL cells. GSEA also revealed activation of the p53 and apoptosis pathways in OT treated cells as well as decreased expression of genes regulating cell cycle progression, metabolism, and DNA repair (**Figures 4A, B**). Moreover, gene ontology analysis showed that genes upregulated by OT treatment are involved in cell differentiation and cell morphogenesis (**Figure 4C**) whereas genes downregulated are involved in ribosome biogenesis, RNA processing/metabolism, and nucleotide synthesis (**Figure 4D**). These results imply impaired synthesis of nucleic acids, RNA maturation, and translation in OT treated cells. Taken together, our RNA-seq data indicate that OT treatment reverses a MYB-driven gene expression program, inhibits cell proliferation, and induces differentiation and apoptosis in T-ALL cells.

### 3.4 Treatment of *MYB*-activated T-ALL cells with OTs results in apoptosis and decreased cell proliferation

**Figure 5:**
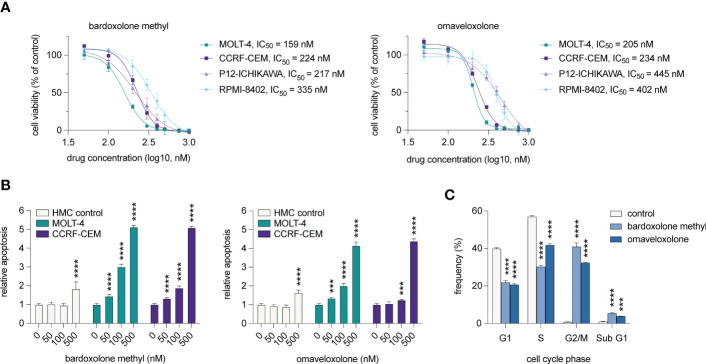
OT treatment leads to G2/M arrest, decreased cell viability, and induction of apoptosis in T-ALL cells. **(A)** Cell viability of T-ALL cells treated with OTs for 72 h. Data points represent the average of three independent experiments. Error bars indicate standard error of the mean. IC_50_ is defined as the drug concentration reducing cell viability by 50%. **(B)** Apoptosis as measured by caspase 3/7 activity after 48 h of treatment with OTs. **(C)** Cell cycle distribution of MOLT-4 cells after 24 h treatment with 250 nM OTs. Error bars indicate standard error of the mean. One-way ANOVA test (*** *P* < 0.001; **** *P* < 0.0001). Data shown represents one of three independent experiments. HMC, human mononuclear cells isolated from a healthy donor.

To investigate if T-ALL cell viability is affected by treatment with OTs, we treated T-ALL cell lines derived from four cases with 50-1000 nM of bardoxolone methyl or omaveloxolone for 72 h (**Figure 5A**). The treatments resulted in a dose-dependent decrease in cell viability at nanomolar concentrations. There was also a significant induction of apoptosis in T-ALL cells after treatment with both drugs (**Figure 5B**). Importantly, HMC control cells were unaffected at low nanomolar concentrations and showed markedly less apoptosis at 500 nM compared with T-ALL cells. Cell cycle analysis showed that OT treated T-ALL cells displayed a marked G2/M arrest (**Figure 5C**). This was in agreement with both OT treatment and *MYB* knockdown causing a decreased expression of the G2 regulating genes *CCNB1* and *CDK1* (**Figures 2C, F**). OT treated cells also displayed a reduced entry into the S phase and an increased accumulation of apoptotic cells in sub G1 (**Figure 5C**). These results, which corroborate our findings from the global gene expression analysis (**Figures 3**, **4**), show that T-ALL cells are sensitive to OT treatment.

### 3.5 OT treatment sensitizes T-ALL cells to chemotherapy

**Figure 6:**
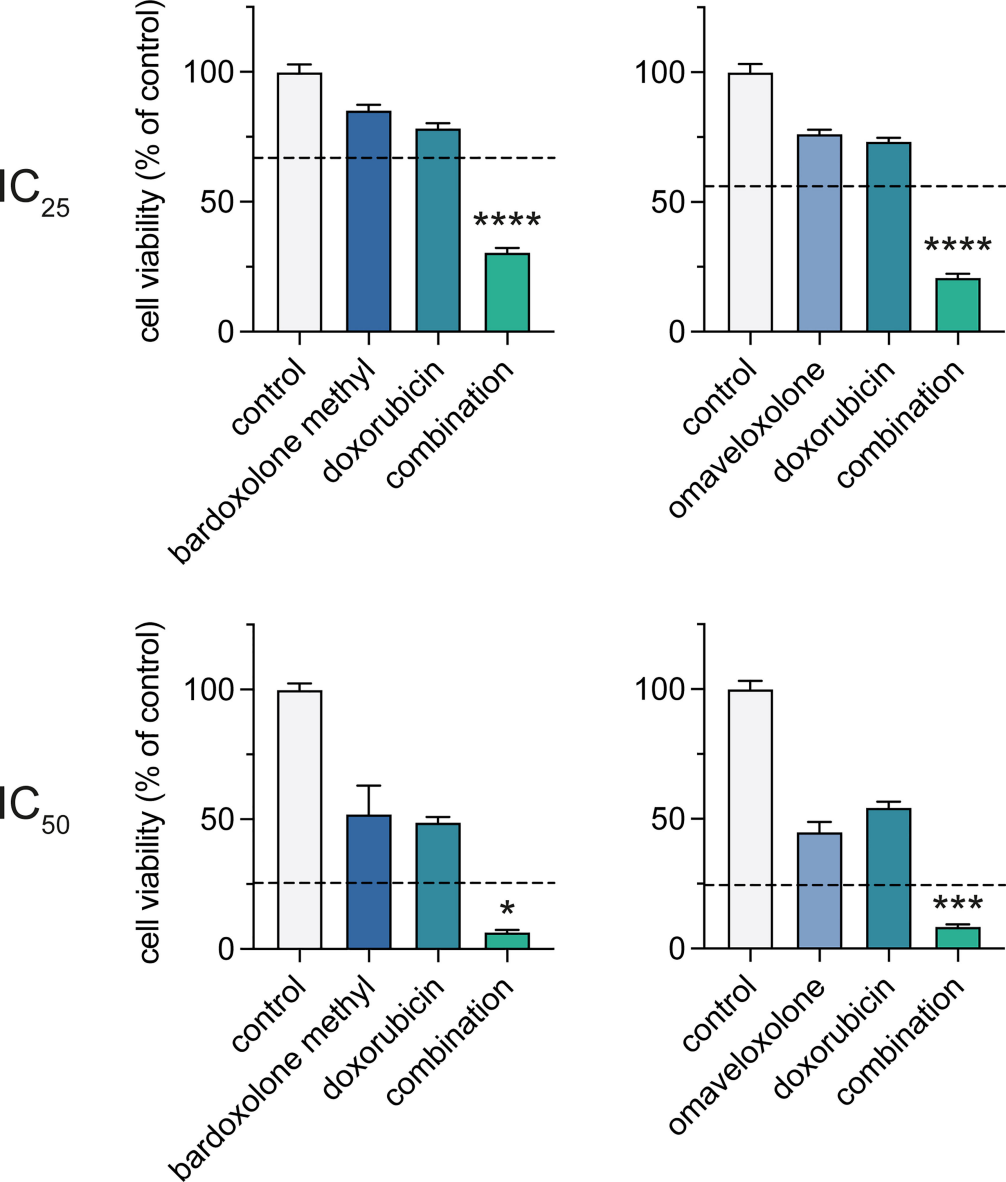
Combined treatment with OTs and doxorubicin leads to a synergistic reduction in cell viability of T-ALL cells. Cell viability of MOLT-4 cells after treatment with IC_25_ and IC_50_ concentrations of bardoxolone methyl or omaveloxolone combined with doxorubicin for 72 h. The expected additive effects of combination treatments, as predicated by Bliss interaction, are indicated with dashed lines. Independent samples t-test (* *P* < 0.05; *** *P* < 0.001; **** *P* < 0.0001). Error bars indicate standard error of the mean. Data shown represents one of three independent experiments.

Since genes involved in DNA repair were downregulated following OT treatment (**Figure 4B**), we tested whether the OTs could synergize with the DNA-damaging agent doxorubicin that is part of the standard therapy of T-ALL. Treatments with bardoxolone methyl or omaveloxolone combined with doxorubicin at IC_25_ and IC_50_ concentrations (**Figure 5A**, **Supplementary Figure S5**) resulted in a significant synergistic decrease in cell viability at both concentrations in MOLT-4 cells (**Figure 6**). These results suggest that synthetic OTs may sensitize T-ALL cells to chemotherapy.

## 4 Discussion

The *MYB* oncogene is an oncogenic driver in the majority of leukemias and encodes a master transcription factor with critical roles in cell proliferation, cell survival, and leukemic stem cell maintenance (4). To find novel inhibitors of *MYB* gene expression, we have now performed a large-scale screening to identify drugs with inhibitory effects on *MYB* mRNA expression in T-ALL cells with constitutive *MYB* gene activation.

Notably, the most potent inhibitors of *MYB* gene expression in our drug screen were bardoxolone methyl and omaveloxolone, two structurally related synthetic OTs that inhibit NF-κB and activate NRF2 signaling (46, 47). Treatment with bardoxolone methyl and omaveloxolone led to downregulation of *MYB* mRNA and protein levels and decreased expression of MYB downstream target genes in a dose-dependent manner. The OTs also negatively affected cell proliferation and viability of T-ALL cells through induction of a marked G2/M arrest, apoptosis, and differentiation. Since both NF-κB pathway inhibition and NRF2 activation failed to downregulate *MYB* gene expression in T-ALL cells, this suggests that the two OT drugs act through alternative mechanisms to inhibit *MYB* activity. Our results indicate that the OTs inhibit one or more positive regulators of *MYB* gene expression in T-ALL cells, possibly independent of NF-κB and NRF2-signaling.

OTs are natural compounds present in a large number of dietary and medicinal plants (48). Synthetic OTs such as bardoxolone methyl and omaveloxolone are generated by chemical modification of oleanolic acid and have anti-inflammatory and anticarcinogenic properties. We have now, for the first time, identified members of this group of compounds as potent inhibitors of *MYB* gene expression. OTs mediate their pharmacological effects in part by interaction with sulfhydryl groups of cysteine residues through Michael addition, yet do not bind non-discriminately to all cysteine-containing proteins (49). Instead, OT binding is markedly influenced by the accessibility of the drug to a specific cysteine residue and the redox potential of the cell. Thus, individual OTs are expected to have different target proteins depending on the cellular context. In addition to targeting the NF-κB and NRF2 pathways, synthetic OTs negatively affect proliferation by inhibiting specific proteins involved in the JAK/STAT and PI3K/AKT/mTOR signaling pathways (49). These pathways may regulate *MYB* expression in T-ALL cells as several JAK/STAT and PI3K/AKT/mTOR inhibitors were among the top hits in our drug screen. Moreover, mTOR signaling has previously been shown to regulate *MYB* expression in normal human T-cells (50). Previous studies have shown that OTs also may induce cell differentiation (49). Similarly, we found that genes involved in positive regulation of differentiation were upregulated in T-ALL cells following treatment with bardoxolone methyl or omaveloxolone. Since the hematopoietic progenitor marker *CD34* (51) was downregulated by both OTs and MYB knockdown, this suggests that the differentiation seen following OT treatment is, at least partly, mediated by decreased MYB expression in T-ALL cells. This conclusion is further supported by studies showing that MYB expression is high in immature and progenitor-like cells and suppress differentiation of both normal and leukemic cells (7, 26).

Bardoxolone methyl is in late clinical stage development for treatment of chronic kidney disease and omaveloxolone recently received FDA approval for treatment of Friedreich’s ataxia. These drugs are currently used in several active and completed clinical trials in which they have demonstrated acceptable adverse effects (https://clinicaltrials.gov). In the present study, the two OTs were active in the low nanomolar range in T-ALL cells whereas normal bone marrow-derived cells were unaffected at these concentrations. Thus, there may be a therapeutic window for these drugs to be repurposed for use in T-ALL patients with acceptable side-effects. Notably, both bardoxolone methyl and omaveloxolone showed a synergistic negative effect on T-ALL cell viability when used in combination with the cytotoxic DNA-damaging agent doxorubicin, which is part of the standard therapy of T-ALL. Since the expression of DNA repair genes was attenuated following treatment, suppression of DNA repair may be part of the underlying mechanism that sensitizes T-ALL cells to combined treatment with OTs and doxorubicin. However, further studies are needed to determine the association between OT treatment, DNA repair, and sensitivity to chemotherapy in T-ALL. It would also be interesting to study the effects of OTs in combination with other drugs such as for example angiogenesis or NOTCH inhibitors (52, 53). Taken together, our studies suggest that bardoxolone methyl and omaveloxolone are new potential drugs for treatment of T-ALL.

Other drugs that we identified with potent inhibitory effects on *MYB* gene expression included several HDACi (e.g. givinostat, panobinostat, quisinostat) and CDKi (e.g. AT7519, SNS-032, p276-00). Previous studies have shown that HDACi (e.g. givinostat and vorinostat, also identified in our screen) downregulate *MYB* gene expression in myeloproliferative neoplasms and myeloid leukemias (36, 39). Here, we identified HDACi as potent inhibitors of *MYB* gene expression also in lymphoid leukemia cells, suggesting a conserved mechanism of *MYB* gene regulation in neoplasms of both the myeloid and lymphoid lineages. Our findings, and those of others (36, 39, 54), thus support the involvement of chromatin remodeling in the regulation of the *MYB* gene. Notably, several CDK9 inhibitors had also inhibitory effects on *MYB* gene expression in our drug screen. The inhibitory effect on *MYB* gene activity by these inhibitors is most likely a result of blocked transcription, since *MYB* gene expression is known to be positively regulated by the transcriptional elongation factor P-TEFb (which includes CDK9) and is inhibited by CDKi (e.g. AT7519 and flavopiridol, also identified in our screen) in breast cancer cells (37, 38). Notably, the plant-derived triterpenoid celastrol was one of the top hits in our screen. Celastrol was previously reported as an inhibitor of MYB transcriptional activity in AML cells (12) and, like the OTs identified here, forms covalent Michael adducts with target proteins (55). This suggests similar mechanisms of *MYB* inhibition by celastrol, bardoxolone methyl, and omaveloxolone. In contrast to the synthetic OTs identified in our screen, celastrol is a natural compound that cause severe side effects, has low bioavailability and poor water solubility, which have hindered its clinical application (56). A number of other MYB inhibitors have been identified with therapeutic effects on AML cells (13–24). It would be interesting to test if these inhibitors also have anti-leukemic effects in MYB-driven T-ALL cells. Only one of these, mebendazole, has to our knowledge been tested and shown effects in T-ALL preclinical models (25). However, many of these inhibitors, unlike the OTs found in our study, are early-stage experimental drugs that have not been tested in humans or show poor pharmacokinetics and/or toxicity. Future studies will evaluate the potential of these inhibitors as new therapies for T-ALL.

Targeting the *MYB* oncogene in a clinical setting will require information about the *MYB* activation status. Since the mechanism of activation of *MYB* varies in T-ALL, analysis of the expression of *MYB* by RT-PCR, RNA-seq or immunohistochemistry are the preferable methods to determine whether the gene is activated or not (57). T-ALL patients with overexpression of *MYB* are thus candidates for MYB inhibitory treatments, whereas those with low or undetectable *MYB* levels are not. *MYB* expression analysis will be crucial for stratification of patients in clinical trials using MYB inhibitors.

A limitation of this study is the lack of testing of bardoxolone methyl and omaveloxolone in *in vivo* T-ALL models and patient primary cells. However, we could demonstrate that normal bone marrow-derived cells are largely unaffected by the drugs compared with T-ALL cells. This is in line with the fact that the drugs show low toxicity in clinical trials (49). Furthermore, the mechanism by which the OTs negatively affect *MYB* expression is unknown. We believe that the drugs may act by similar mechanisms as celastrol, which blocks MYB activity in AML (12), but they may also regulate *MYB* expression through inhibition of for example JAK/STAT and/or PI3K/AKT/mTOR signaling. OTs do not fit the single-target paradigm and has multiple molecular targets, yet they show low cytotoxicity in normal cells (49). This multifunctionality may be an advantage in order to potentiate treatment and to avoid drug resistance. In T-ALL, multiple driver events, in addition to MYB activation, is involved in the pathogenesis of the disease, including *NOTCH1* mutations and loss of *CDK2NA* (11). This strongly implies that inhibition of MYB must be combined with targeting of additional pathways for successful treatment.

In conclusion, we have identified several novel suppressors of *MYB* gene expression by large-scale drug screening of cultured T-ALL cells with constitutive *MYB* gene activation. In particular, the structurally related OTs bardoxolone methyl and omaveloxolone demonstrated potent inhibitory effects on *MYB* gene expression and MYB downstream targets, and induced apoptosis in *MYB* gene activated T-ALL cells. Notably, these drugs sensitized T-ALL cells in culture to DNA-damaging chemotherapy. Our results indicate that both bardoxolone methyl and omaveloxolone may synergize with the standard treatment of T-ALL but also have the potential to improve current therapies of other MYB-driven malignancies.

## Data availability statement

The datasets presented in this study can be found in online repositories. The names of the repository/repositories and accession number(s) can be found below: https://www.ncbi.nlm.nih.gov/sra/PRJNA811771.

## Author contributions

MA conceptualized, designed, and supervised the study. PN, TT, AA, AF, and MA performed experiments. PN, TT, AA, AF, GS, and MA analyzed data. GS and MA provided project resources. MA drafted the manuscript. All authors reviewed and edited the manuscript. All authors contributed to the article and approved the submitted version.
