# Supplementary figures and images for: Synthetic oleanane triterpenoids suppress MYB oncogene activity and sensitize T-cell acute lymphoblastic leukemia cells to chemotherapy
Source: Front Oncol. 2023 Apr 3;13:1126354. doi: 10.3389/fonc.2023.1126354 (PMC10106619; doi:10.3389/fonc.2023.1126354)

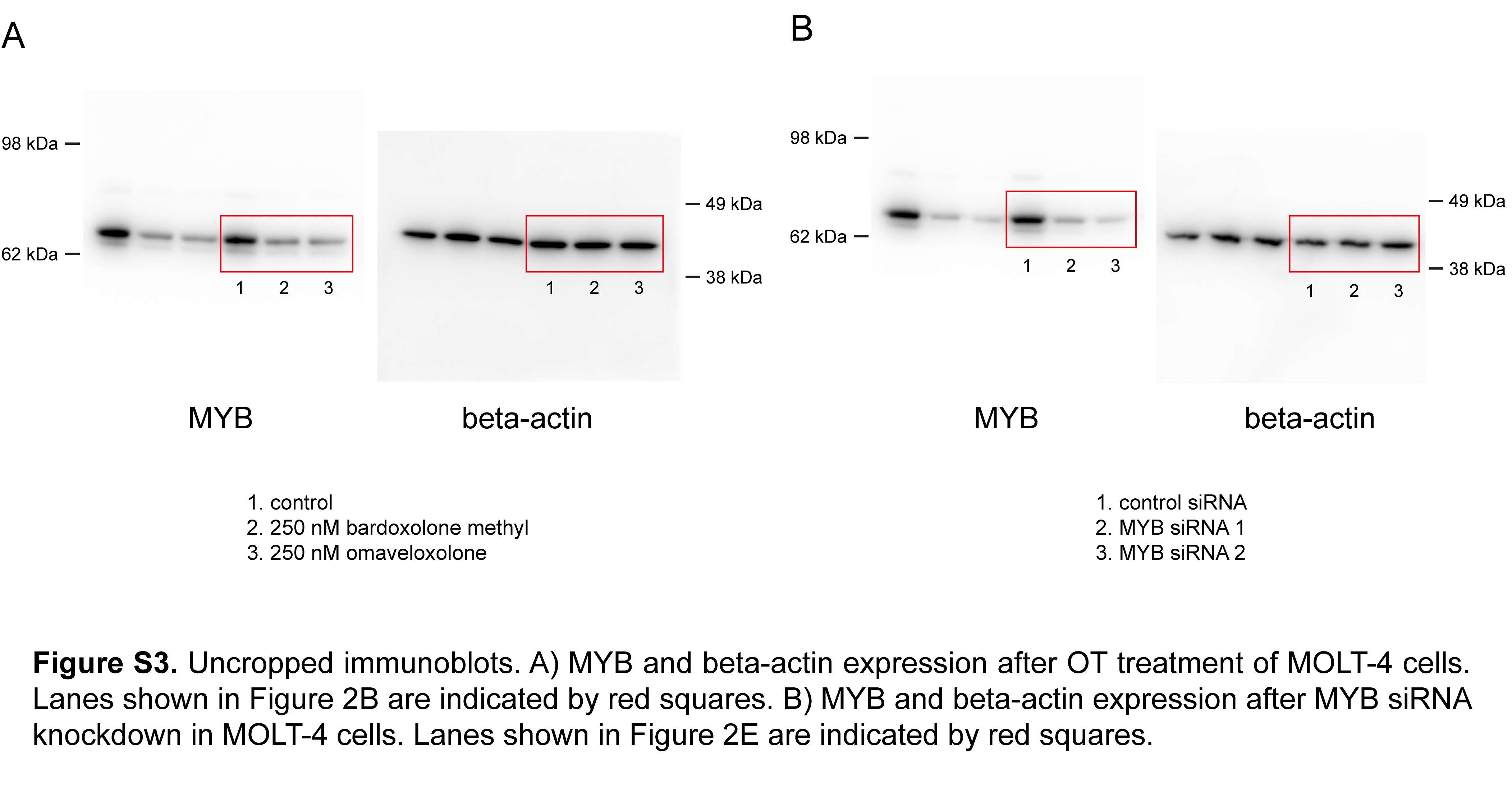

Supplement: Supplementary file 1 [file DataSheet_1.zip › Figure S3_600DPI.tif]

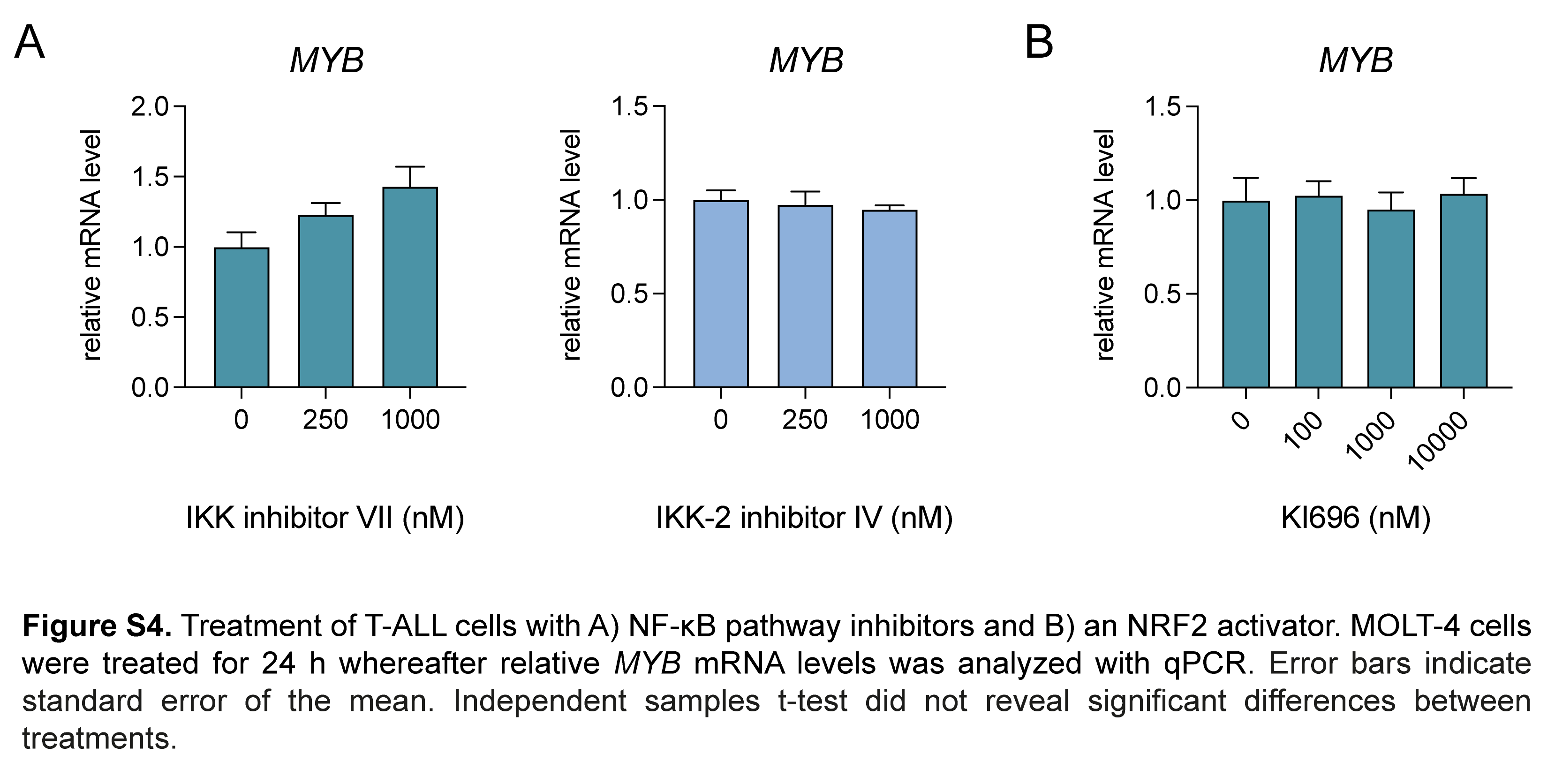

Supplement: Supplementary file 1 [file DataSheet_1.zip › Figure S4_600DPI.tif]

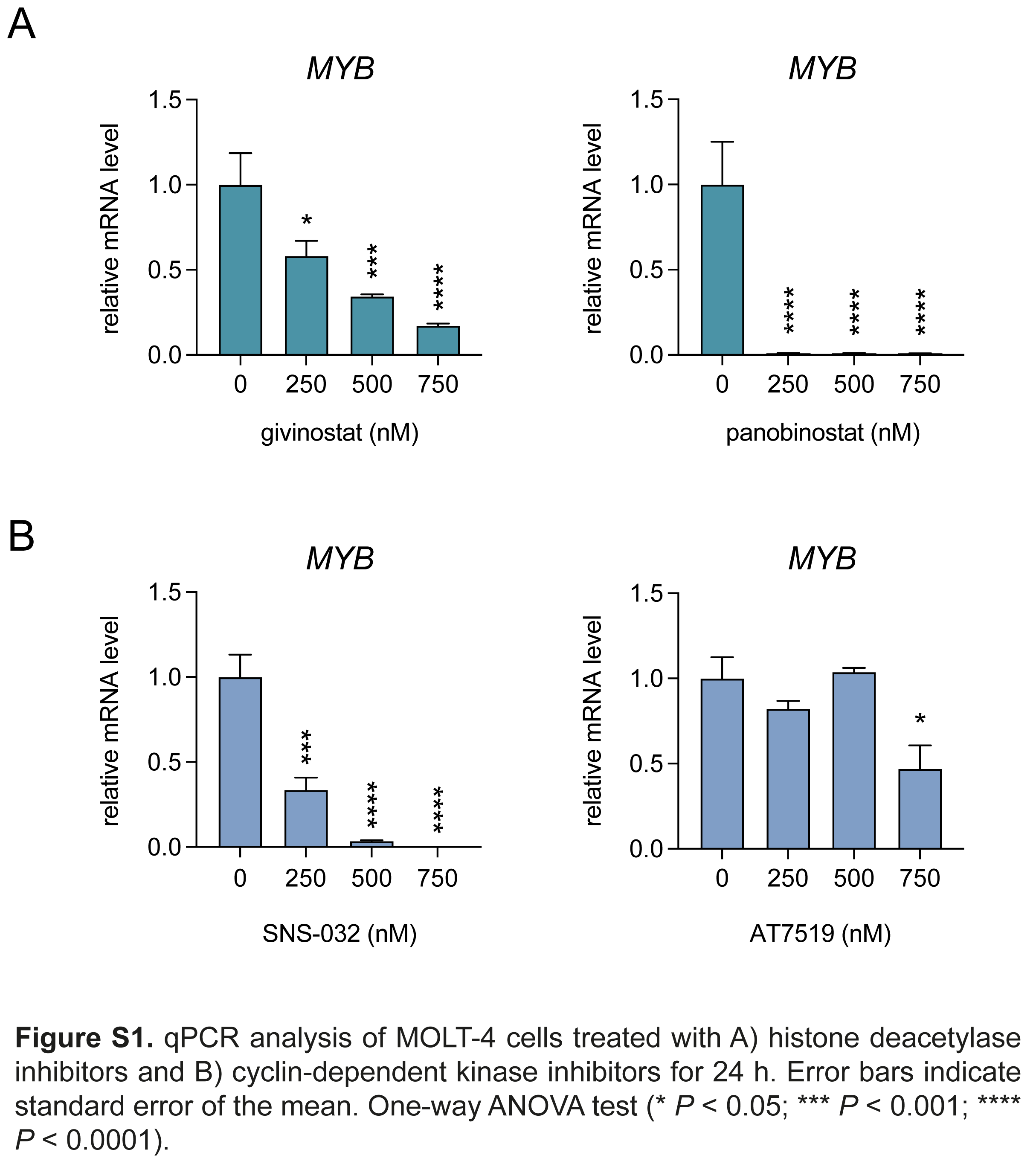

Supplement: Supplementary file 1 [file DataSheet_1.zip › Figure S1_600DPI.tif]

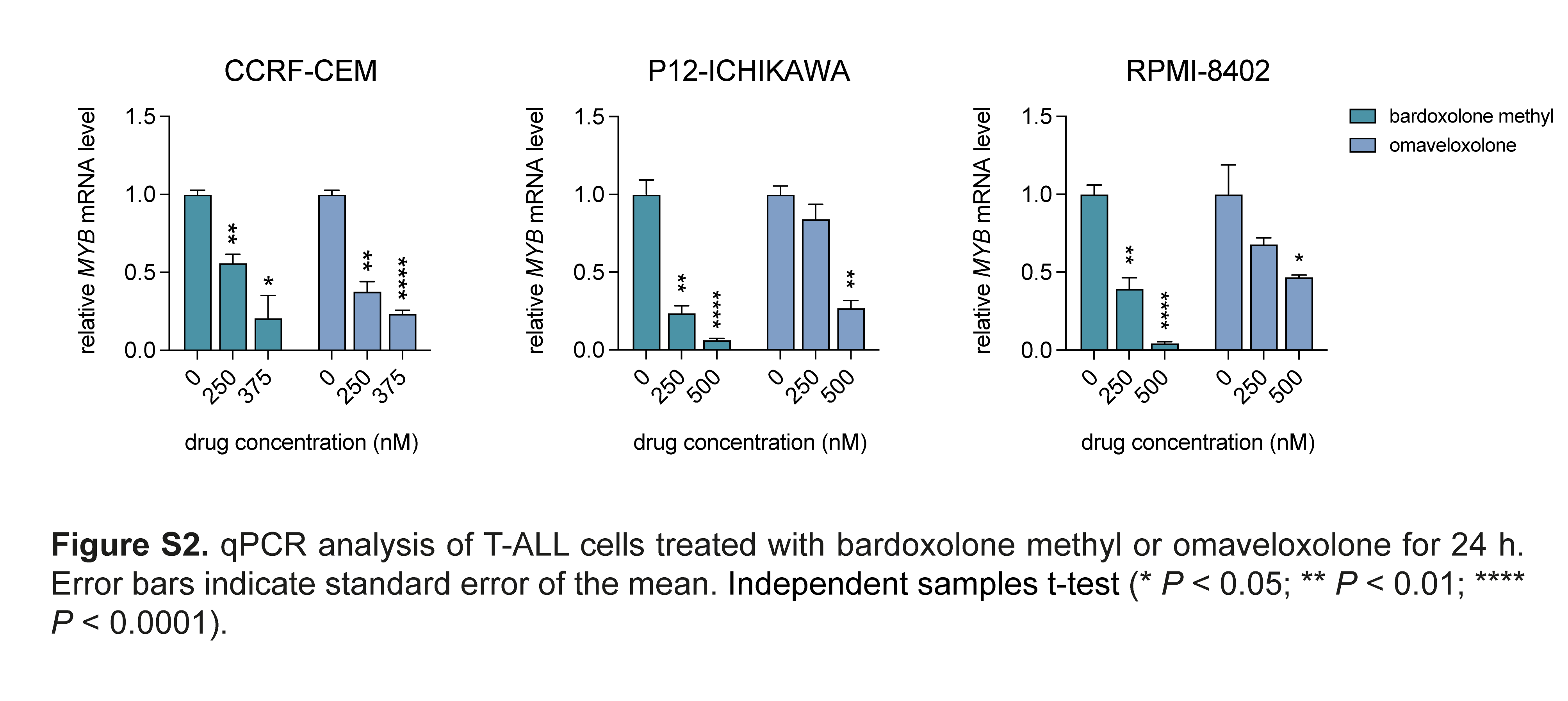

Supplement: Supplementary file 1 [file DataSheet_1.zip › Figure S2_600DPI.tif]

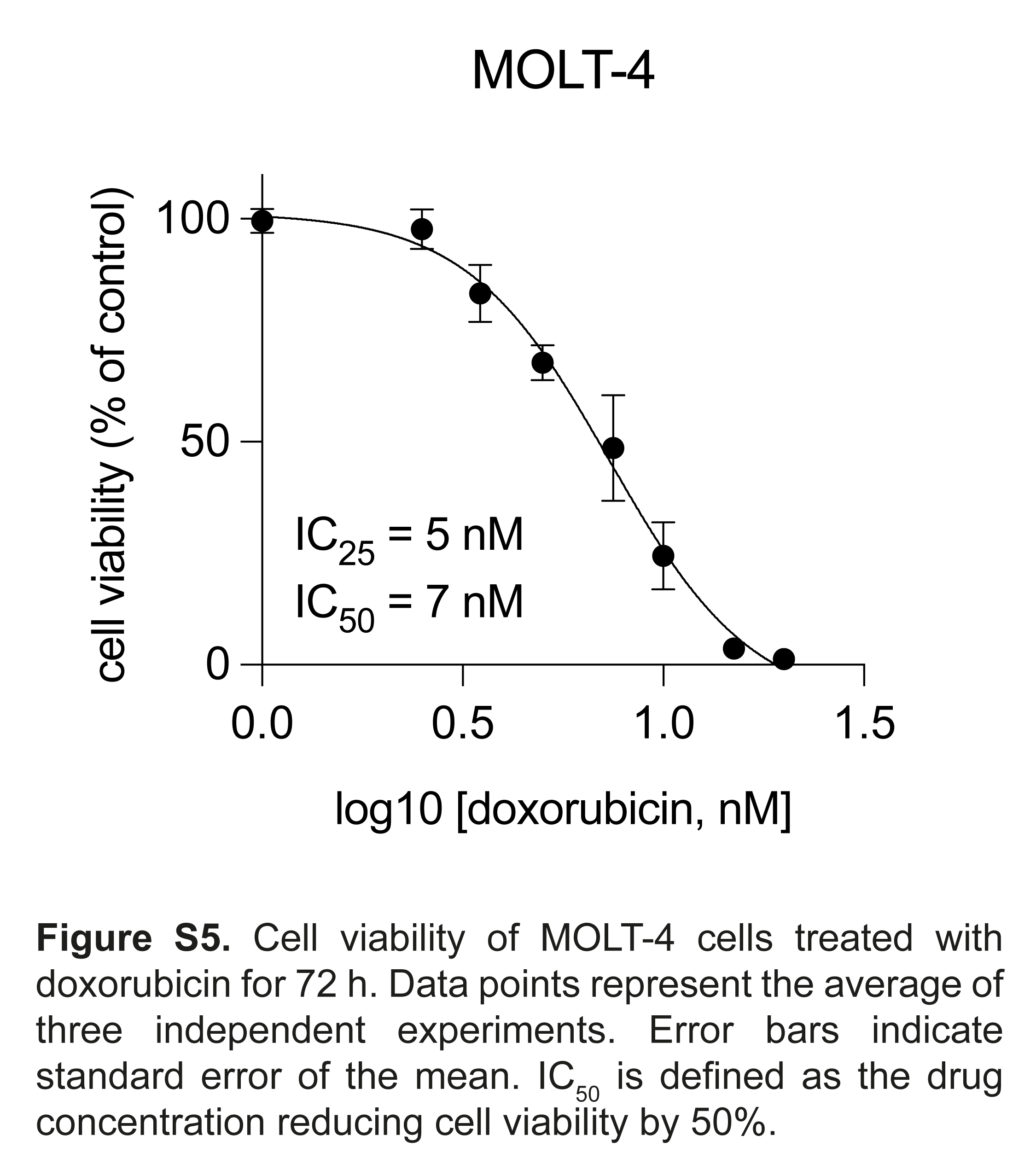

Supplement: Supplementary file 1 [file DataSheet_1.zip › Figure S5_600DPI.tif]
